# A single nucleotide polymorphism in kidney anion exchanger 1 gene is associated with incomplete type 1 renal tubular acidosis

**DOI:** 10.1038/srep35841

**Published:** 2016-10-21

**Authors:** Takumi Takeuchi, Mami Hattori-Kato, Yumiko Okuno, Atsushi Kanatani, Masayoshi Zaitsu, Koji Mikami

**Affiliations:** 1 Department of Urology, Japan Organization of Occupational Health and Safety, Kanto Rosai Hospital, 1-1 Kizukisumiyoshi-cho, Nakahara-ku, Kawasaki 211-8510, Japan; 2 Division of Molecular Pathology, Institute of Medical Science, The University of Tokyo, 4-6-1 Shirokanedai, Minato-ku, Tokyo 108-8639, Japan

## Abstract

Various conditions including distal renal tubular acidosis (dRTA) can induce stone formation in the kidney. dRTA is characterized by an impairment of urine acidification in the distal nephron. dRTA is caused by variations in genes functioning in intercalated cells including SLC4A1/AE1/Band3 transcribing two kinds of mRNAs encoding the Cl^−^/HCO3^−^ exchanger in erythrocytes and that expressed in α-intercalated cells (kAE1). With the acid-loading test, 25% of urolithiasis patients were diagnosed with incomplete dRTA. In erythroid intron 3 containing the promoter region of kAE1, rs999716 SNP showed a significantly higher minor allele A frequency in incomplete dRTA compared with non-dRTA patients. The promoter regions of the kAE1 gene with the minor allele A at rs999716 downstream of the TATA box showed reduced promoter activities compared that with the major allele G. Patients with the A allele at rs999716 may express less kAE1 mRNA and protein in the intercalated cells, developing incomplete dRTA.

An upper urinary tract stone is a very common disease, and the annual incidence is estimated to be 203.1 in Japan^1^ and 457.02 per 100,000 citizens in South Korea^2^. Various conditions including distal renal tubular acidosis (type 1 RTA, dRTA) can induce stone formation in the kidney^3^. dRTA is characterized by an impairment of urine acidification due to the dysfunction of α-intercalated cells in the distal nephron despite a relatively maintained glomerular filtration rate^4^. Urinary stones formed in dRTA patients usually include a calcium phosphate component. Potassium citrate therapy is useful for the prevention of calcium stone formation in acidotic patients^5^,^6^.

Acquired dRTA can be observed in patients with Sjögren syndrome, systemic lupus erythematosus, obstructive uropathy, acute tubular necrosis, chronic pyelonephritis, renal transplantation, analgesic nephropathy, sarcoidosis, idiopathic hypercalciuria, primary parathyroidism, and drug-induced nephropathy brought about by lithium, amphotericin, cyclosporine, and tacrolimus^3^,^4^. dRTA is also caused by variations in genes functioning in intercalated cells, i.e., cytosolic carbonic anhydrase 2 (CA2)^7^,^8^, ATP6V1B1^9^,^10^,^11^ encoding the B1 subunit of H^+^-ATPase, ATP6V0A4 encoding the A4 subunit of H^+^-ATPase, and SLC4A1/AE1/Band3^12^,^13^,^14^ encoding the Cl^−^/HCO3^−^ exchanger. The former three show autosomal recessive inheritance and the latter is autosomal dominant and autosomal recessive. Anion exchanger protein 1 (AE1) is a dimeric glycoprotein^15^,^16^ with 14 transmembrane domains^17^ and it participates in the regulation of the intracellular pH by Cl^−^/HCO3^−^ exchange across the cell membrane. In the kidney, it is located at the basolateral membrane of α-intercalated cells and functions in the secretion of H^+^ into the tubular lumen in cooperation with H^+^-ATPase and H^+^/K^+^-ATPase at the apical membrane^4^,^18^.

SLC4A1/AE1/Band3 is a gene spanning 19.757 kb of genomic DNA situated in chromosome 17, q21-22. The gene consists of twenty exons and transcribes two kinds of mRNAs utilizing different promoters encoding the Cl^−^/HCO3^−^ exchanger in erythrocytes (eAE1) and that expressed in α-intercalated cells in the kidney (kAE1). The promoter for kAE1 is located in erythroid intron 3, and so the kAE1 transcript lacks exons 1 through 3 of the eAE1 transcript^19^.

We investigated the capacity for urine acidification by administering an acid-loading test to patients who had previously undergone interventional treatment for adult-onset upper urinary tract stones. Also, the kAE1 gene was analyzed for those showing impaired urine acidification to assess whether they had some genetic variations, as they may possibly have ones different from those found in complete dRTA patients diagnosed in infancy with more severe symptoms.

## Materials and Methods

### Acid-loading test

Sixty-eight unrelated Japanese patients (57 males and 11 females, age: 57.1 ± 12.6 years [range: 29–83]) who had previously undergone transurethral lithotripsy and/or extracorporeal shockwave lithotripsy for the treatment of upper urinary tract stones were investigated regarding their capacity for renal tubular acidification during hospitalization. A total of 35.3% of the participants were recurrent stone formers and 48.8% had multiple or staghorn stones. With data on the analysis of stone components available in 32 out of the 68 cases, urinary stones of 14 patients (43.8%) contained a calcium phosphate component. Exclusion criteria were severe renal dysfunction (eGFR <45 mL/min/1.73 m^2^) and liver dysfunction. Alkaline replacement therapy, if administered (n = 22), was withdrawn 2 weeks prior to the study. No participants had skeletal abnormality, growth retardation, deafness, neurological disorder, or severe hematological disorder. After fasting for at least 8 hours, 0.1 g/kg body weight of NH_4_Cl was orally taken over a period of 2 hours together with 500 mL of water. Before and hourly following the ingestion of NH_4_Cl, urine was collected for six hours. The pH of each urine sample was measured immediately by a pH meter. Before and three hours after the ingestion of NH_4_Cl, venous blood gas was measured.

### Analysis of AE1 gene

Genomic DNA was extracted from whole blood collected in EDTA containing tubes using a kit (DNA Extractor® WB Kit, Wako, Osaka, Japan). For genomic DNAs of patients who showed urine acidification abnormality, the SLC4A1/AE1/Band3 gene was amplified in 5 fragments encompassing all exons except exon 1 (non-coding exon), i.e., from exon 2 to exon 20, using sense/antisense pairs of primers previously described^20^. PCRs were performed using a thermal cycler (Thermal cycler Wako WK-0518, Wako, Osaka, Japan) in a 50 μL volume with 0.3 μM of sense and antisense primers, 2 μL of MgSO_4_, 1 unit of KOD plus polymerase (TOYOBO, Osaka, Japan), and the buffer supplied with the enzyme as follows: PCR-1 and PCR-2: 94 °C for 2 min, 36 cycles (98 °C for 10 sec for denaturing, 68 °C for 3 min for annealing and extension) of 2 step PCR with 4% dimethyl sulfoxide (DMSO), PCR-3 and PCR-4: as above without DMSO, PCR-5: 94 °C for 2 min, 36 cycles (98 °C for 10 sec for denaturing, 64 °C for 30 sec for annealing, 68 °C for 3 min for extension) of 3 step PCR without DMSO. Amplified PCR products (10 μL) were resolved by electrophoresis in 1% agarose gel. PCR products were directly sequenced with sequencing primers previously reported^20^ in addition to the primers 5′-CAGGACTTAAGTGCTGGAGA-3′, 5′-CACTATGCCCTGGACTATGC-3′ and 5′-CAGCTCTGACCCTGTCTC-3′ for the sequencing of PCR-1, PCR-2, and PCR-3, respectively.

In order to further analyze nucleotide sequences of erythroid introns 3, 7, and 17 of all participants in the acid-loading test regardless of the urine acidification capacity (n = 13 for incomplete dRTA and n = 29 for non-dRTA), PCR products amplified by pairs of sense/antisense primers were directly sequenced. Primers for amplification were 5′-GGAGAATCTGGAGCAGGAGG-3′/5′-CCCACAAGCCCCTCATTTCT-3′ for intron 3 (428 bp), 5′-GAGGCCCCTCTTGGAAATGA-3′/5′-GGTGTGGTAGTCTGTGGCTG-3′ for intron 3 (542 bp), 5′-GAAGCCTGCAGTCCTGACAC-3′/5′-ACCAACGTGGCCTCTGAATC-3′ for intron 7 (407 bp), and 5′-CAAAGCCAGCACCCCAGG-3′/5′-TCAGTCATGGAGCAGCTGAG-3′ for intron17 (501 bp). Sequencing primers were 5′-GGAGGAATATGAAGACCCAG-3′, 5′-GGAGGGGTGGGAATATAAGG-3′, 5′-GATCCTTCACAGCCTCTGC-3′, and 5′-GTCAAAGAGCAGCGGATCAG-3′, respectively. PCRs were performed in a 50-μL volume with 0.3 μM sense and antisense primers, 2 μL of MgSO_4_, 4% DMSO (for erythroid introns 3 and 7), and 1 unit of KOD plus polymerase as follows: 94 °C for 2 min, 40 cycles (98 °C for 10 sec for denaturing, 60 °C for 30 sec for annealing, 68 °C for 30 sec for extension) of 3 step PCR.

### Analysis of the B1 subunit of the H^+^-ATPase and CA2 genes

Exons of the B1 subunit of H^+^-ATPase and CA2 genes of incomplete dRTA patients in this study were amplified using previously reported primers^10^,^21^ located in the flanking introns of each exon. PCRs were basically performed in a 50-μL volume with 0.3 μM sense and antisense primers, 2 μL of MgSO_4_, 4% DMSO, and 1 unit of KOD plus polymerase as follows: 94 °C for 2 min, 40 cycles (98 °C for 10 sec for denaturing, 60 °C for 30 sec for annealing, 68 °C for 30 sec for extension) of 3 step PCR. Annealing temperatures for exons 2, 3, and 4 of CA2 were 55 °C. The DMSA concentration for exon 1 of CA2 was 8%. Direct sequencing was done with PCR primers.

### Promoter reporter assay

Intron 3 of the AE1 gene of an incomplete dRTA case and a non-dRTA control were amplified by two pairs of sense/antisense primers: 5′-GCGCGAGCTCGCCGTTCTCCCCTCAATTCT-3′/5′- GCGCAAGCTTACCTCTGAGACCAGACTCCC-3′ and 5′- GCGCGAGCTCGAGGCCCCTCTTGGAAATGA-3′/5′-GCGCAAGCTTCCCTTCTCTCCTCCCACCTG-3′, in a 50-μL volume with 0.3 μM sense and antisense primers, 2 μL of MgSO_4_, 4% DMSO, and 1 unit of KOD plus polymerase as follows: 94 °C for 2 min, 2 cycles (98 °C for 10 sec, 60 °C for 30 sec, 68 °C for 30 sec) of 3 step PCR followed by 38 cycles (98 °C for 10 sec, 68 °C for 30 sec) of 2 step PCR. Sense and antisense primers were attached with SacI and HindIII restriction sites at the 5-prime ends, respectively. The former PCR product (PCR-L) of 553 base pairs contained major alleles at SNP sites of rs16940585 and rs16940582, while the latter one (PCR-S) of 151 base pairs did not contain those SNP sites. The PCR-L had a TATA box, GT boxes, GATA sites, CACCC boxes, a CCAAT box, and an AP2 binding site, while the PCR-S had only a TATA box and GT boxes^19^. PCR products from the incomplete dRTA case (Case 59) had the minor allele A at the SNP site of rs999716, while those from the control had the major allele G there. PCR products, PCR-L and PCR-S, were inserted into the SacI/HindIII cloning site of pGL4.17 [luc2/Neo] reporter vector (Promega, Madison, WI, USA) following digestion with SacI and HindIII, leading to the formation of recombinant constructs, L-pGL4.17 and S-pGL4.17. L(S)-pGL4.17 vectors from the incomplete dRTA case and the control were named dRTA-L(S)-pGL4.17 and Control-L(S)-pGL4.17, respectively.

Madin-Darby canine kidney (MDCK) cells and Human Embryonic Kidney 293 (HEK 293) cells were plated in a 96-well plate at cell densities of 0.3 × 10^4^ cells/well and 2 × 10^4^ cells/well, respectively, and were grown overnight in DMEM with 10% FBS to approximately 50% confluency. pGL4.17, dRTA-L(S)-pGL4.17, and Control-L(S)-pGL4.17 vectors were used to transfect the MDCK and HEK293 cells. The DNA mix for transfection was prepared in DMEM and consisted of 100 ng of the test plasmid and 1 ng of the pNL1.1.TK[Nluc/TK] control vector (Promega, Madison, WI) expressing NanoLuc^®^ luciferase under the control of the herpes simplex virus thymidine kinase promoter serving as an internal control to normalize firefly luciferase activity. The transfection was carried out using Lipofectamine 2000 (Invitrogen Corporation, Carlsbad, CA, USA). After 24 hours, the firefly luciferase activity was determined using the Nano-Glo® Dual-Luciferase® Reporter (NanoDLR™) Assay System (Promega Corporation, Madison, WI) in the GloMax^®^ Discover (Promega Corporation, Madison, WI). Each sample was triplicated and experiments were conducted three times. The normalized strength of the firefly luciferease activity was calculated by the firefly luciferease activity of each well divided by the NanoLuc^®^ luciferase activity of the same well.

### Statistics

Values in Table 1 were analyzed by the two-tailed unpaired t-test except for the male:female ratio and the multiple stone rate. The male:female ratio and multiple stone rate were analyzed by the two-tailed Fisher’s exact test. Allele frequencies in Table 2 were analyzed between incomplete dRTA cases and non-dRTA cases for each SNP with two-tailed Fisher’s exact test. Additionally, Firth logistic regression was applied to SNPs showing significance by two-tailed Fisher’s exact test in order to delineate which SNP(s) independently indicated incomplete dRTA. The minimun urine pH after acid-loading was analyzed for the left and right in each panel by the two-tailed unpaired t-test. Promoter activities were compared by the Bonferroni post-hoc test.

### Study approval

The Ethical Committee of Kanto Rosai Hospital approved the experiments. All experiments were performed in accordance with relevant guidelines and regulations, including any relevant details. Written informed consent was received from participants prior to inclusion in the study.

## Results

### Acid-loading test

**Figure 1:**
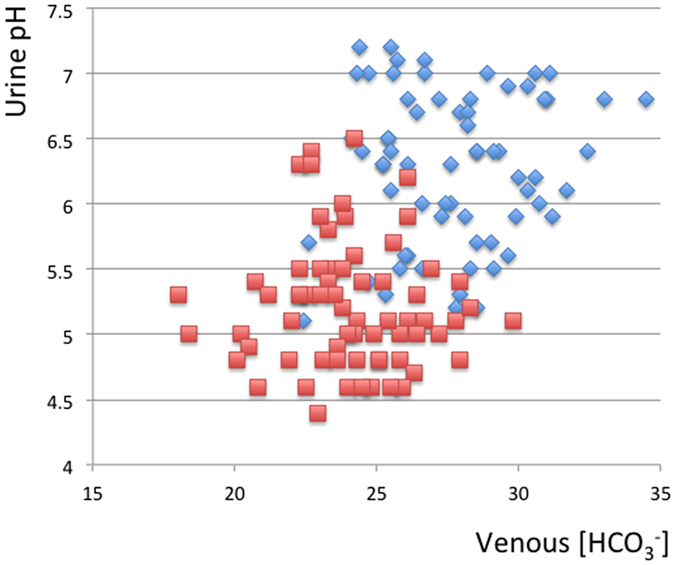
Scatter plots of urine pH and venous [HCO3^−^] (mEq/L) before and after acid-loading, blue boxes: urine pH and venous [HCO3^−^] (mEq/L) before acid-loading, red boxes; minimum urine pH and venous [HCO3^−^] (mEq/L) three hours after acid-loading.

Figure 1 indicates scatter plots of urine pH and plasma bicarbonate in venous blood gas (venous [HCO3^−^]). No patients in this study showed spontaneous metabolic acidosis, venous [HCO3^−^] lower than 20.0 mEq/L, before acid-loading concomitant with a urine pH above 5.5. Thus, complete dRTA patients were not identified. After acid-loading, venous [HCO3^−^] was lowered by 3.7 ± 2.5 mEq/L and it became lower than 20 mEq/L in 2 cases (2.9%). The urine pH became lower than 5.5 in 51 (75.0%) of the participants, but not in 17 (25.0%). Therefore, these 17 patients were diagnosed with incomplete type 1 RTA, because the urine pH was constantly above 5.5 after acid-loading. Factors potentially inducing acquired dRTA were not clear in patients diagnosed with incomplete dRTA in this study.

The characteristics of the 68 patients are described in Table 1. Patients diagnosed with incomplete dRTA were older than non-dRTA patients. The percentage of females was higher in incomplete dRTA than in non-dRTA. The multiple stone rate was marginally higher in incomplete dRTA than in non-dRTA. Four incomplete dRTA cases (23.5%), but none of the non-dRTA cases, showed staghorn renal stones (p = 0.0029 by Fisher’s exact test). Baseline serum Na, Cl, Ca, IP, venous [HCO3^−^], eGFR, and the urine Ca/Cr ratio were not significantly different between non-RTA and incomplete dRTA. The baseline serum K was slightly lower and baseline urine pH was higher in incomplete dRTA than in non-dRTA.

### Analysis of AE1 gene

In thirteen incomplete dRTA cases that did not show a lowering of the urine pH below 5.5 after acid-loading as well as three non-dRTA controls, the AE1 gene was analyzed. Table 3 summarizes variations in exons. Exon 4 of an incomplete dRTA case (Case 11) showed heterozygous missense mutation (rs5035), which results in the Darmstadt D38A amino acid variant of erythroid AE1 (eAE1), but is a regulatory SNP for kAE1. In addition, a control non-dRTA urolithiasis patient and an incomplete dRTA patient (Case 29) had a heterozygous silent mutation in exon 12 (rs13306781). Exon 19 of another incomplete dRTA (Case 2) had heterozygous G to A substitution, which does not change the amino acids of kAE1 and has not been registered in the dbSNP database.

**Figure 2:**
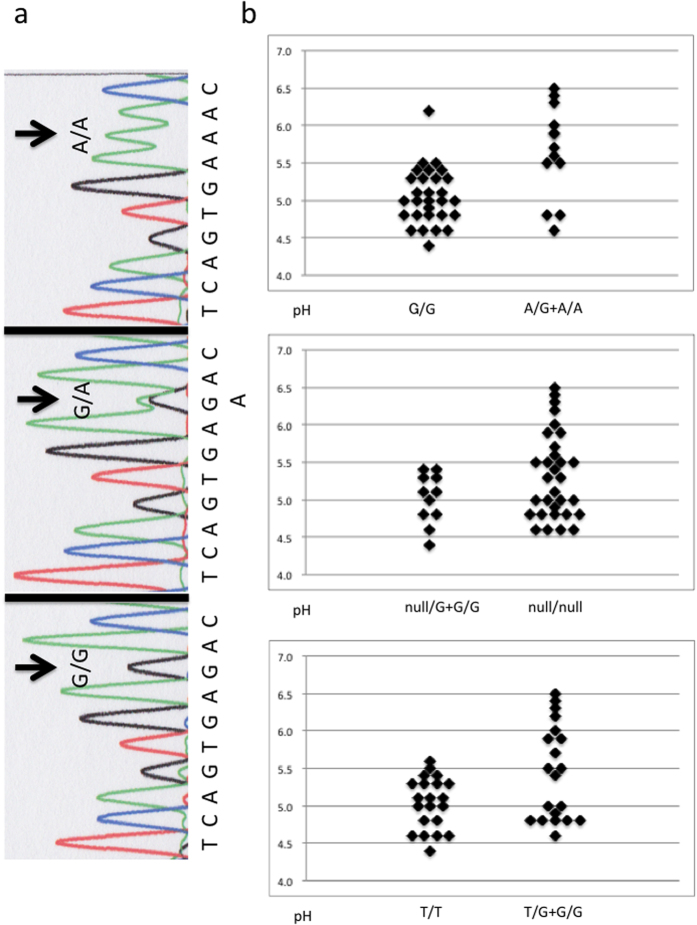
(**a**) Sequencing of rs999716, G/G; homozygous G/G alleles, G/A; heterozygous G/A alleles, A/A; homozygous A/A alleles. (**b**) Minimum urine pH after acid-loading, Top; rs999716 (5.06 ± 0.37 vs. 5.65 ± 0.62*), Middle; rs45538331 (5.02 ± 0.33 vs. 5.32 ± 0.57), Bottom; rs2857078 (5.04 ± 0.34 vs. 5.44 ± 0.63*), pH (left column vs. right column, mean ± standard deviation), *p < 0.05 by the unpaired student t test.

In introns of incomplete dRTA cases, minor alleles of registered SNPs were identified in introns 3, 7, and 17, as shown in Table 2. In intron 3 containing the promoter region of kAE1, rs999716 SNP (Fig. 2a) showed a significantly higher minor allele frequency in patients with impaired urine acidification compared with those without it. The frequency of homozygous A/A plus heterozygous A/G alleles at rs999716 in incomplete dRTA was also significantly higher than that in non-dRTA. Other SNPs in intron 3 did not reveal a significant difference in the minor allele frequency between patients with and those without impaired urine acidification. In introns 7 and 17, allele frequencies of rs2857082, rs45538331, and rs2857078 showed significant differences between incomplete dRTA and non-dRTA. Null/null alleles at rs45538331 in intron 7 and G/G plus T/G alleles at rs2857078 in intron 17 were more frequently identified in incomplete dRTA cases.

With multivariate analysis using Firth logistic regression, only homozygous A/A plus heterozygous A/G alleles at rs999716 in erythroid intron 3 showed significance (Table 2). Patients with homozygous G/G alleles at rs999716 showed a significantly lower minimum urine pH after acid-loading, while those with homozygous A/A and heterozygous A/G alleles showed a higher minimum urine pH, mostly above 5.5, as shown in Fig. 2b. Values of the baseline urine pH before acid-loading were not significantly different between patients with minor allele A at rs999716 and those without it (6.44 ± 0.57 vs. 6.29 ± 0.53, respectively, p = 0.43 by the unpaired t-test). The multiple stone rates were comparable between patients with minor allele A at rs999716 and those without (69.2 vs. 41.4%, respectively, p = 0.1809 by Fisher’s test). Four patients (30.8%) with minor allele A at rs999716, but none without it, showed staghorn renal stones (p = 0.0064 by Fisher’s exact test).

Collectively, minor allele A at SNP rs999716 accounts for 76.9% of incomplete dRTA cases with upper urinary tract stones. It also accounted for 14.7% of upper urinary tract stones in this study, if incomplete dRTA is assumed to be the main cause of urolithiasis.

### Analysis of exons of the B1 subunit of the H^+^-ATPase gene

Among the 13 incomplete dRTA patients, heterozygous c.27T > C variation (rs17853498) in exon 1 of the B1 subunit of the H^+^-ATPase gene of Case 63, heterogenous and homogeneous c.1002C > T variation (rs2072462) in exon 10 of all 13 cases, heterogenous c.1023C > T variation (rs117826071) in exon 10 of Case 35, and homogeneous c.1320T > G variation (rs147250093) in exon 13 of Case 59 were identified. All variations in exons of the B1 subunit of the H^+^-ATPase gene were silent mutations that do not change amino acid sequences.

### Analysis of exons of the CA2 gene

Among the 13 incomplete dRTA patients, heterozygous (n = 1) and homozygous (n = 9) c.259T > C variation (rs703) that does not change amino acid sequences was identified in exon 6 of the CA2 gene.

### Promoter reporter assay

**Figure 3:**
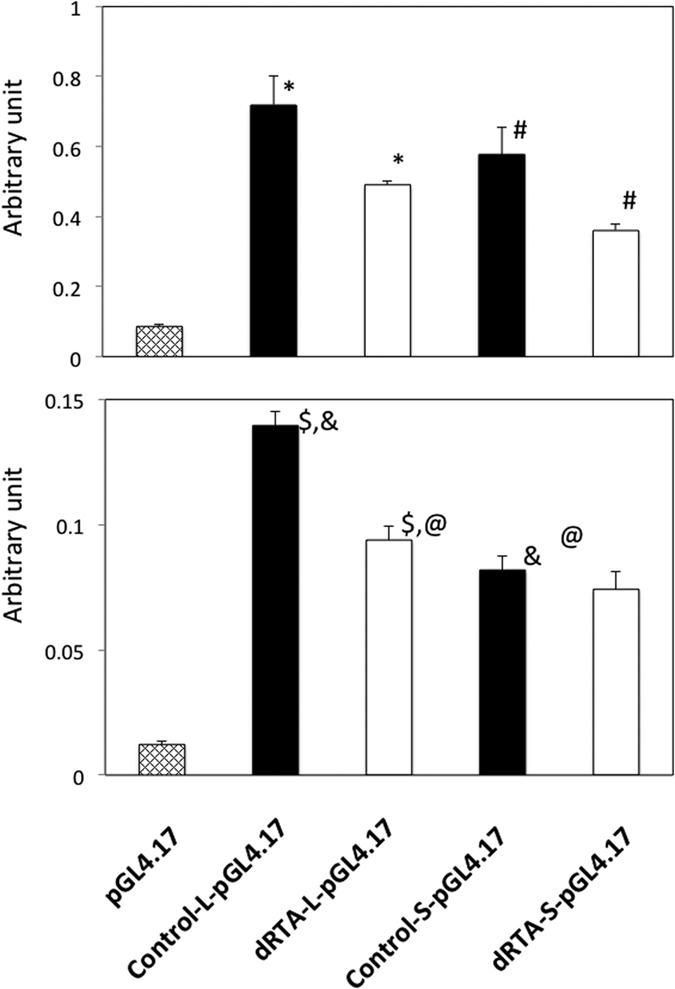
Promoter activity of kAE1 in MDCK and HEK 293 cells, vertical axis; firefly luciferase activity under the test promoter sequence divided by NanoLuc^®^ luciferase under the control of the herpes simplex virus thymidine kinase promoter, Upper panel; MDCK cells, *p = 0.003, ^#^p = 0.004, Lower panel; HEK 293 cells, ^$^p < 0.001, &p = p < 0.001, ^@^p < 0.001 by the Bonferroni post-hoc test, Bars; standard deviations.

As shown in Fig. 3, the promoter activity of every transfected recombinant reporter vector was enhanced compared with that of pGL4.17 without the insertion of a promoter region in both MDCK and HEK 293 cells. In both cell types, recombinant reporter vectors with the minor allele A at rs999716 from an incomplete dRTA patient, dRTA-L-pGL4.17 and dRTA-S-pGL4.17, showed reduced promoter activities compared with the corresponding reporter vectors with the major allele G, Control-L-pGL4.17, and Control-S-pGL4.17, respectively.

## Discussion

Variations in the AE1 gene have been extensively investigated in dRTA patients, but most involved amino acid changes in cases that are found in infancy and with phenotypes more serious than urolithiasis alone, such as hemolytic anemia, severe metabolic acidosis, rickets, osteomalacia, massive nephrocalcinosis, renal failure, and growth retardation^18^. Genetic variants in the kAE1 gene possibly identified in adult-onset urolithiasis patients have been postulated to be milder and even without amino acid changes. In the present study, urolithiasis patients did not show severe metabolic acidemia before acid-loading. After acid-loading, one quarter were identified as incomplete dRTA cases, with the percentage being similar to that among recurrent calcium-containing renal stone formers in previous reports^22^,^23^. Upper urinary tract stones that occurred in incomplete dRTA patients tended to be multiple and occasionally form staghorn renal stones in this study compared with those in non-dRTA ones.

In erythroid intron 3 of the AE1 gene containing the promoter region for transcribing the kAE1 transcript, some minor alleles of registered SNPs were identified in incomplete dRTA cases in the present study. These SNPs in intron 3 can be regarded as regulatory rather than intronic for kAE1. Considering that the frequencies of minor allele A as well as homozygous A/A plus heterozygous A/G alleles at the rs999716 site in incomplete dRTA cases were significantly higher than those in non-dRTA ones, possession of the A allele at rs999716 may be causative of incomplete dRTA. Clearly, homozygous A/A and heterozygous A/G alleles at rs999716 were distinct from homozygous G/G alleles in the impairment of lowering the urine pH after acid-loading. Therefore, it is strongly suggested that rs999716 is involved in the pathogenesis of incomplete dRTA causing adult-onset urolithiasis.

Rs999716 is located in 39 base pairs downstream of the TATA box in the promoter region of the kAE1 gene. The TATA box is a type of promoter DNA sequence where transcription factors can bind and recruit RNA polymerase, leading to the synthesis of RNA from DNA. Therefore, polymorphism in the sequences flanking the TATA box can influence the affinity of TATA-binding protein and level of transcription^24^. Thus, single nucleotide substitution at the rs999716 site has the potential to induce changes in the efficient transcription of kAE1 mRNA. Actually, the promoter region with the minor allele A at rs999716 showed lower promoter activity than that with the major allele G based on the promoter reporter assay. Patients with A allele at rs999716 may express lower amount of kAE1 mRNA and kAE1 protein in the intercalated cells in the distal nephron, and so they have impaired urine acidification leading to incomplete dRTA.

There are exceptions. One patient with homozygous G/G alleles showed a minimum urine pH of 6.2 after acid-loading, and incomplete dRTA in this case may have been due to a different cause than a lower amount of kAE1 protein. In the present study, there were no mutations that change amino acid sequences in the exons of the B1 subunit of H^+^-ATPase and CA2 genes. Two patients with heterozygous A/G alleles and one patient with homozygous A/A alleles showed a minimum urine pH below 5.0 after acid-loading. In these cases, kAE1 protein expression may have remained normal for unknown reasons, or molecules other than kAE1 may have functioned sufficiently to lower the urine pH.

Two unrelated Indian patients showing combined ovalocytic hemolytic anemia and complete dRTA with homozygous A858D mutations of exon 19 of the AE1/SLC4A1 gene were reported^20^. Interestingly, these two patients had homozygous A/A alleles at rs999716. Heterozygous individuals with the dominant A858D mutation were diagnosed with incomplete dRTA as they cannot acidify their urine after a frusemide/fludrocortisone test^25^. The A858D mutant retains some of the wild-type protein in the endoplasmic reticulum. Then, the amount of functional protein at the cell surface in heterozygotes is diminished^26^. c.-119G > A for kAE1 at rs999716 may also have contributed to the aggravation of dRTA to some extent in these reported Indian cases. c.2116 + 315T > G for kAE1 at rs2857078 in intron 17 detected in incomplete RTA cases in the present study was also homozygous in the two Indian patients cited above. The A allele of rs2857078 was associated with preeclampsia^27^.

The minor allele frequency (A) of rs999716 was reported to be 34.1% by the 1,000 Genomes Project^28^. Considering the relatively high frequency of the minor allele at rs999716, “acquired” dRTA occurring in patients with, for example, Sjögren syndrome may be potentially in accordance with c.-119G > A of the kAE1 gene in addition to the non-genetic, generalized causes of renal tubular damage such as autoantibodies. kAE1 expressions in kidney biopsies were reported to be reduced in patients with Sjögren syndrome concomitant with dRTA^29^,^30^.

One variation in exon 4 of the AE1 gene was identified in an incomplete dRTA case. That missense substitution (rs5035) caused a heterozygous D38A amino acid change in eAE1. D38A was previously reported^31^, but its clinical significance remains unknown. The promoter for the kAE1 transcript is located in intron 3 of the AE1 gene. Then, D38A does not substitute an amino acid in the kAE1 protein in the kidney, and it is interpreted as a variation in the 5′ untranslated region for kAE1. Silent mutations that do not change amino acids existed in exon 12 and exon 19 in a non-dRTA control patient and incomplete dRTA cases. It was recently suggested that even silent mutations have the potential to cause changes in protein translation efficiency and protein folding^32^,^33^, but it may not apply to these cases as there was no abnormality in urine acidification in the non-dRTA patient. Collectively, variations in exons identified in this study were sporadic and do not cause amino acid changes. Thus, these variations are suggested to have little or no linkage with the induction of incomplete dRTA.

There are exogenous, endogenous, and environmental causes of urolithiasis. In addition to allelic variation at rs999716, a high-calorie diet, obesity, dehydration, and anatomical abnormality in the urinary tract may facilitate the formation of renal stones. As high as 19 to 42% of urolithiasis patients are reported to have a family history, with a higher rate in women^34^,^35^,^36^. Women may be more influenced by stone-biased genetic variations, such as rs999716. In conclusion, a single nucleotide polymorphism at rs999716 flanking the TATA box in the kAE1 gene causes incomplete type 1 renal tubular acidosis, leading to the formation of upper urinary tract stones.

## Additional Information

**How to cite this article**: Takeuchi, T. *et al.* A single nucleotide polymorphism in kidney anion exchanger 1 gene is associated with incomplete type 1 renal tubular acidosis. *Sci. Rep.* **6**, 35841; doi: 10.1038/srep35841 (2016).

## Figures and Tables

**Table 1:** Patient characteristics and biochemical data.

	Non-dRTA (n = 51)	Incomplete dRTA (n = 17)
Age	54.7 ± 12.3	64.2 ± 11.4*
Male: Female	46 : 5	11 : 6**
Multiple stone rate	41.2%	70.6%^#^
eGFR	72.8 ± 17.2	68.8 ± 11.8
Serum Na	140.4 ± 2.3	140.4 ± 1.4
Serum K	4.3 ± 0.3	4.0 ± 0.5*
Serum Cl	104.0 ± 2.1	104.3 ± 1.4
Serum Ca	9.4 ± 0.3	9.2 ± 0.3
Serum IP	3.1 ± 0.4	3.2 ± 0.6
HCO3^−^ (VBG)	27.8 ± 2.4	26.7 ± 1.9
Urine pH	6.17 ± 0.58	6.64 ± 0.43*
Urine Ca/Cr	0.13 ± 0.11	0.15 ± 0.14

*p < 0.05 vs. non-dRTA by the unpaired *t*-test, **p < 0.05 by Fisher’s exact test, ^#^p = 0.053 by Fisher’s exact test, VBG: venous blood gas, incomplete dRTA: urine pH above 5.5 after acid-loading, Data represent the mean ± standard deviation except for the male:female ratio.

**Table 2:** Univariate analysis by two-tailed Fisher’s Exact Test.

SNP ID (variation of kAE1)	Major	Minor	MAF (%)	p-value			p-value
Intron 3							
rs777861687 (c.-676G > T)	G	T		0.3095	GG	GG + GT	0.3095
incomplete dRTA	25	1	3.8		12	1	
non-dRTA	58	0	0.0		29	0	
rs16940585 (c.-457C > T)	C	T		1.0000	CC	TT + CT	0.6966
incomplete dRTA	22	4	15.4		9	4	
non-dRTA	49	9	15.5		23	6	
rs16940582 (c.-440G > A)	G	A		0.3263	GG	AA + GA	0.6953
incomplete dRTA	24	2	7.7		11	2	
non-dRTA	48	10	17.2		22	7	
rs999716 (c.-119G > A)	G	A		0.0001	GG	AA + GA	0.0001
incomplete dRTA	13	13	50.0		3	10	
non-dRTA	54	4	7.1		26	3	
rs2074106 (c.-90 + 92C > A)	C	A		0.2367	CC	AA + CA	0.7225
incomplete dRTA	11	15	57.7		3	10	
non-dRTA	34	24	41.1		9	20	
rs398119837 (c.-89-118_−89-117insG)	null	G		0.4005	null null	GG + G null	0.7387
incomplete dRTA	22	4	15.4		9	4	
non-dRTA	43	15	26.8		18	11	
rs13306782 (c.-90 + 174G > A)	G	A		0.2251	GG	AA + GA	0.7004
incomplete dRTA	24	2	7.7		11	2	
non-dRTA	57	1	1.8		28	1	
Intron 7							
rs2857082 (c.414 + 86G > A)	G	A		0.0625	AA	GG + GA	0.2319
incomplete dRTA	18	8	25.0		1	12	
non-dRTA	27	31	53.5		8	21	
rs45538331 (c.415-101_415-100insG)	null	G		0.0040	null/null	null/G + GG	0.0093
incomplete dRTA	26	0	0.0		13	0	
non-dRTA	44	14	24.1		18	11	
Intron 17							
rs2857078 (c.2116 + 315T > G)	T	G		0.0028	TT	TG + GG	0.0063
incomplete dRTA	11	13	54.2		2	10	
non-dRTA	47	11	19.0		19	10	
Major: major allele,	Minor: minor allele, MAF: minor allele frequency						
Multivariate analysis predicting incomplete dRTA using Firth logistic regression.							
Parameters	Odds ratio			95%CI	p-value		
A/G and A/A at rs999716	36.19			1.03–10.24	0.003		
Null/null at rs45538331	5.22			−1.70–7.10	0.344		
T/G and G/G at rs2857078	0.73			−4.80–2.88	0.853		
Female Sex	14.2			0.29–8.03	0.026		
Age (60 yrs ≦ )	2.43			−1.15–3.54	0.383		

The Firth logistic regression was used because there was complete separation at rs45538331.

**Table 3:** Exonal kAE1 gene variation analysis in incomplete dRTA patients.

Exons	SNP ID	Variation	Type	Patients
Exon 4	rs5035	c.113A > C for eAE1	p.D38A for eAE1	1 incomplete dRTA
		c.-83A > C for kAE1	regulatory SNP for kAE1	
Exon 12	rs13306781	c.1314C > T for eAE1	silent mutation	1 incomplete dRTA & 1 non-dRTA
		c.1119G > A for kAE1		
Exon 19	not registered	c.2487G > A for eAE1	silent mutation	1 incomplete dRTA
		c.2292G > A for kAE1		
